# Supplementary material for: Phenol-Soluble Modulin α3 Stimulates Autophagy in HaCaT Keratinocytes
Source: Biomedicines. 2023 Nov 10;11(11):3018. doi: 10.3390/biomedicines11113018 (PMC10669503; doi:10.3390/biomedicines11113018)
Supplement: Supplementary file 1 [file biomedicines-11-03018-s001.zip › biomedicines-2609739-supplementary.pdf]

Supplementary Materials

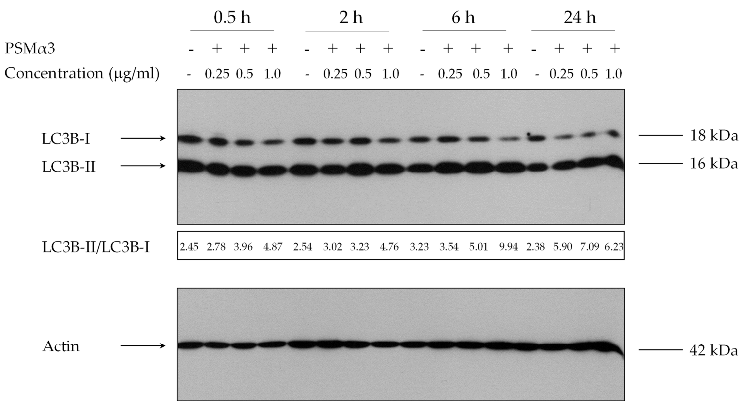

Figure S1. The kinetics of endogenous LC3B-II expression in PSMα3-treated HaCaT keratinocytes

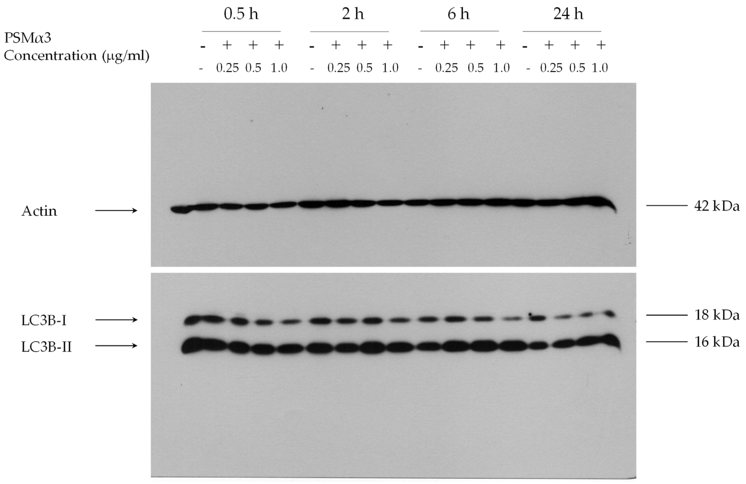

The uncropped western blot images used for Figure S1.

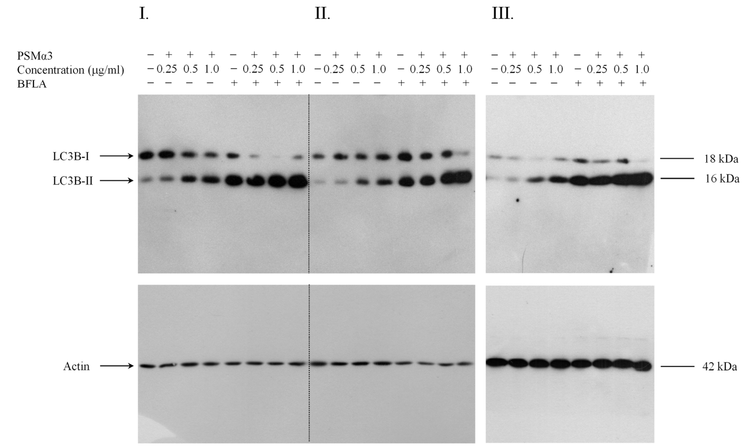

Figure S2. PSMα3 stimulates the autophagic flux.

Western blot images used to calculate the fold changes of LC3B-II shown in Figure 1.

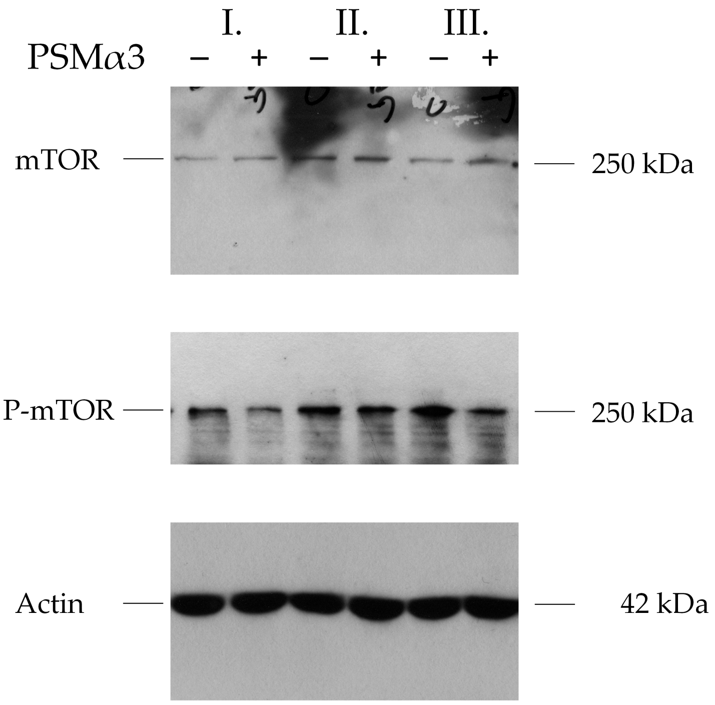

Figure S3: PSMα3 alters the levels of mTOR and phospho-mTOR (S2448) levels in HaCaT keratinocytes. Western blot images used to calculate the fold changes of mTOR and phospho-mTOR (S2448) shown in Figure 5C-F.

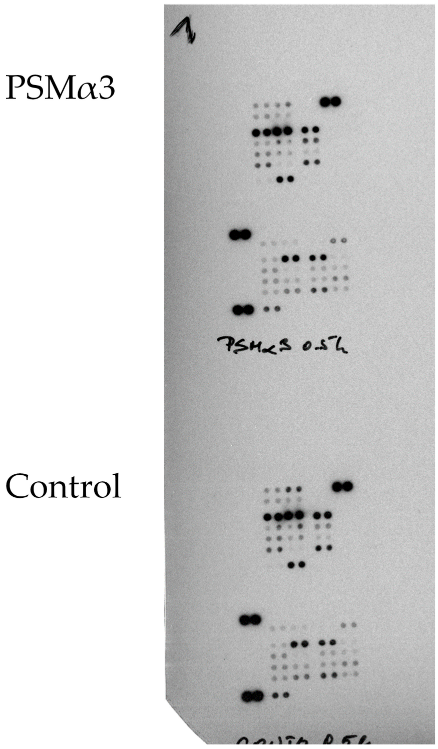

Figure S4: PSMα3 alters the phospho-kinase array profile of HaCaT keratinocytes. Uncropped image of the phospho-kinase array used to create Figure 5A and 5B.

Table S1. List of the detected phospho-proteins by using the Proteome ProfilerTM human phospho-kinase array kit.

Membrane/Coordinate	Target/Control	Phosphorylation Site
**A-A1, A2**	Reference Spot	-
**B-A11, A12**	Akt 1/2/3	T308
**B-A13, A14**	Akt 1/2/3	S473
**B-A17, A18**	Reference Spot	-
**A-B3, B4**	CREB	S133
**A-B5, B6**	EGF R	Y1086
**A-B7, B8**	eNOS	S1177
**A-B9, B10**	ERK1/2	T202/Y204, T185/Y187
**B-B11, B12**	Chk-2	T68
**B-B13, B14**	c-Jun	S63
**A-C3, C4**	Fgr	Y412
**A-C5, C6**	GSK-3α/β	S21/S9
**A-C7, C8**	GSK-3β	S9
**A-C9, C10**	HSP27	S78/S82
**B-C11, C12**	p53	S15
**B-C13, C14**	p53	S46
**B-C15, C16**	p53	S392
**A-D3, D4**	JNK 1/2/3	T183/Y185, T221/Y223
**A-D5, D6**	Lck	Y394
**A-D7, D8**	Lyn	Y397
**A-D9, D10**	MSK1/2	S376/S360
**B-D11, D12**	p70 S6 Kinase	T389
**B-D13, D14**	p70 S6 Kinase	T421/S424
**B-D15, D16**	PRAS40	T246
**A-E3, E4**	p38α	T180/Y182
**A-E5, E6**	PDGF Rβ	Y751
**A-E7, E8**	PLC-γ1	Y783
**A-E9, E10**	Src	Y419
**B-E11, E12**	PYK2	Y402
**B-E13, E14**	RSK1/2	S221/S227
**B-E15, E16**	RSK1/2/3	S380/S386/S377
**A-F3, F4**	STAT2	Y689
**A-F5, F6**	STAT5a/b	Y694/Y699
**A-F7, F8**	WNK1	T60
**A-F9, F10**	Yes	Y426
**B-F11, F12**	STAT1	Y701
**B-F13, F14**	STAT3	Y705
**B-F15, F16**	STAT3	S727
**A-G1, G2**	Reference Spot	-
**A-G3, G4**	β-Catenin	-

**A-G9, G10**	PBS (Negative Control)	-
**B-G11, G12**	STAT6	Y641
**B-G13, G14**	HSP60	-
**B-G17, G18**	PBS (Negative Control)	-
